# *Bacillus coagulans* MA-13: a promising thermophilic and cellulolytic strain for the production of lactic acid from lignocellulosic hydrolysate

**DOI:** 10.1186/s13068-017-0896-8

**Published:** 2017-09-07

**Authors:** Martina Aulitto, Salvatore Fusco, Simonetta Bartolucci, Carl Johan Franzén, Patrizia Contursi

**Affiliations:** 1 Dipartimento di Biologia, Università degli Studi di Napoli Federico II, Naples, Italy; 2 Division of Industrial Biotechnology, Department of Biology and Biological Engineering, Chalmers University of Technology, 412 96 Gothenburg, Sweden

**Keywords:** Lactic acid, *Bacillus coagulans*, Thermophilic, Fermentation, Robustness, Cellulolytic enzymes, Wheat straw hydrolysate, Enzymes secretion

## Abstract

**Background:**

The transition from a petroleum-based economy towards more sustainable bioprocesses for the production of fuels and chemicals (circular economy) is necessary to alleviate the impact of anthropic activities on the global ecosystem. Lignocellulosic biomass-derived sugars are suitable alternative feedstocks that can be fermented or biochemically converted to value-added products. An example is lactic acid, which is an essential chemical for the production of polylactic acid, a biodegradable bioplastic. However, lactic acid is still mainly produced by *Lactobacillus* species via fermentation of starch-containing materials, the use of which competes with the supply of food and feed.

**Results:**

A thermophilic and cellulolytic lactic acid producer was isolated from bean processing waste and was identified as a new strain of *Bacillus coagulans*, named MA-13. This bacterium fermented lignocellulose-derived sugars to lactic acid at 55 °C and pH 5.5. Moreover, it was found to be a robust strain able to tolerate high concentrations of hydrolysate obtained from wheat straw pre-treated by acid-catalysed (pre-)hydrolysis and steam explosion, especially when cultivated in controlled bioreactor conditions. Indeed, unlike what was observed in microscale cultivations (complete growth inhibition at hydrolysate concentrations above 50%), *B. coagulans* MA-13 was able to grow and ferment in 95% hydrolysate-containing bioreactor fermentations. This bacterium was also found to secrete soluble thermophilic cellulases, which could be produced at low temperature (37 °C), still retaining an optimal operational activity at 50 °C.

**Conclusions:**

The above-mentioned features make *B. coagulans* MA-13 an appealing starting point for future development of a consolidated bioprocess for production of lactic acid from lignocellulosic biomass, after further strain development by genetic and evolutionary engineering. Its optimal temperature and pH of growth match with the operational conditions of fungal enzymes hitherto employed for the depolymerisation of lignocellulosic biomasses to fermentable sugars. Moreover, the robustness of *B. coagulans* MA-13 is a desirable trait, given the presence of microbial growth inhibitors in the pre-treated biomass hydrolysate.

**Electronic supplementary material:**

The online version of this article (doi:10.1186/s13068-017-0896-8) contains supplementary material, which is available to authorized users.

## Background

Given the finite nature of fossil-derived fuels and chemicals as well as their negative impact on the environment, it is important to develop technologies for the use of alternative and more eco-friendly feedstocks [1]. For instance, lignocellulosic biomasses from municipal, agricultural and forestry origins are continuously being generated by several anthropic activities [2]. Therefore, they are relatively inexpensive and abundant carbon sources that can be exploited for the production of biofuels and biochemicals, without giving a net contribution to the emissions of CO_2_ into the atmosphere. Conversely, they can negatively contribute to the environmental pollution if not properly disposed of, recycled or valorised [3].

Currently, the global renewable biochemicals market is growing in size and importance, with polylactic acid (PLA) being one of the main shares among bioplastics [4]. PLA is a polymer of d- and l-lactic acid (LA), the latter of which can be obtained by fermentation of renewable feedstocks [5, 6]. Currently, several carbohydrates and nitrogenous materials are commercially used for the production of lactic acid, e.g. sucrose-containing syrups, juices and molasses; lactose from whey, maltose, glucose and mannitol [7]. Moreover, several *Lactobacillus* species have been recently employed for the production of LA from starch-containing materials. However, the use of such biomasses may compete with the supply of foods and feeds [8]. To overcome this conflict, non-food sources of fermentable sugars, i.e. lignocellulosic biomasses, are suitable alternatives [9]. The major component of these residues is cellulose (35–50%), which occurs together with hemicellulose (20–40%) and lignin (10–30%) at varying compositions that depend on the plant source [10]. Cellulose is a homopolymer consisting of β-1,4-linked d-glucose monomers. Unlike starch, it is not directly accessible as a carbon and energy source for the majority of microorganisms.

In nature, biodegradation of lignocellulose is an extremely slow and complex process that involves the concerted action of many microbial decomposers [11]. However, a more effective deconstruction process has been set up at industrial scale and includes three steps: (1) pre-treatment [12]; (2) enzymatic or chemical saccharification and (3) fermentation [13]. In this context, a major bottleneck is represented by the formation of toxic compounds during chemical and thermal pre-treatments [14]. These inhibitory compounds are classified into three major groups: furaldehydes (e.g. furfural and 5-hydroxymethylfurfural), weak acids (e.g. acetic acid, formic acid and levulinic acid) and phenolics (e.g. vanillin, syringaldehyde and coniferyl aldehyde) [15]. Such chemicals hamper microbial growth and affect the fermentation fitness, thus profoundly impacting the economy of the whole process [16]. One strategy to remove the inhibitors is to include a detoxification step [17] but that, in turn, can lead to an increase of production costs [18]. In order to overcome this drawback, an alternative approach is to exploit the natural and/or induced tolerance of fermenting microorganisms (bacteria and yeasts) [16, 19]. In this perspective, it is important to isolate and characterise robust microorganisms for the production of eco-friendly chemicals, such as lignocellulose-based LA for PLA manufacturing [9, 20].

Besides being employed for fermentation, some fungal and bacterial strains are also sources of carbohydrate-active enzymes [21]. The use of biocatalysts for the saccharification of lignocellulosic biomass represents a more sustainable approach than the chemical counterparts [22]. Moreover, if the reaction conditions of both pre-treatment and saccharification are taken into account (i.e. temperature, pH, pressure and presence of inhibitory chemicals), thermostable enzymes are appropriate candidates [23]. Indeed, their operational stability at high temperature allows reducing the enzymes loading for the saccharification and, in turn, makes the production process more economically feasible [24]. In this regard, bacteria of the genus *Bacillus* are well-known to secrete various hydrolytic enzymes such as proteases, amylases and cellulases [25–27]. Therefore, thermophilic *Bacillus* strains might represent reservoirs of novel and robust cellulolytic enzymes.

The saccharification and fermentation phases can be carried out sequentially in a set-up known as separate hydrolysis and fermentation (SHF). Here, the plant cell wall polymers are first hydrolysed by lignocellulolytic enzymes to monomeric sugars which are then fermented by the microbial cells in a separate process [28]. However, the enzymes can be inhibited by the high concentrations of the hydrolysed products (monomeric sugars) achieved during saccharification [29]. For this reason, simultaneous saccharification and fermentation (SSF) has been developed to combine these two processes in the same batch, in order to alleviate the enzymatic inhibition [30]. Recently, consolidated bioprocessing (CBP), a one-step conversion of lignocellulosic biomass into the desired final product, is becoming a commercially attractive alternative [31]. In CBP, a native or genetically engineered microbial strain is used both for enzyme production, leading to hydrolysis of the complex carbohydrates, and at the same time, for fermentation of the released sugars into valued-added products [31, 32].

Here, we report the isolation and characterisation of a new thermophilic and cellulolytic *Bacillus coagulans* strain (MA-13), which is able to (1) secrete cellulolytic enzymes, (2) ferment lignocellulose-derived sugars to lactic acid and (3) withstand the toxicity of inhibitors present in the liquid fraction (hydrolysate) of wheat straw pre-treated by acid-catalysed hydrolysis and steam explosion. The above-mentioned features, together with the possibility to implement new traits through genetic engineering of *B. coagulans* [33], make this microorganism a potential workhorse for the production of lactic acid from lignocellulosic raw materials.

## Methods

### Isolation and screening of cellulolytic microorganisms

The biological sample used in this study was in the form of an agricultural residue from a canned beans manufacturing process. In order to isolate cellulolytic microorganisms, a few grams of this waste residue were resuspended in 100 ml of sterile double-distilled water (ddH_2_O), placed in a 250-ml Erlenmeyer flask with a long neck and incubated at 60 °C with a shaking rate of 180 rpm for 30 min, using a MaxQTM 4000 Benchtop Orbital Shaker (Thermo Scientific). The waste suspension was serially diluted (tenfold) using sterile ddH_2_O and 150 μl from each dilution was spread on plates of solid carboxymethyl-cellulose (CMC; Sigma-Aldrich) containing screening medium (CMC-SM, Additional file 1: Table S1). After overnight incubation at 60 °C in a dry oven, microbial colonies appeared on the surface of the plates. Hundreds of these colonies were picked using sterile needles, transferred into tubes containing liquid CMC-SM and incubated at 60 °C with a shaking rate of 180 rpm. Microbial growth was spectrophotometrically monitored at an optical density of 600 nm (OD_600_) over a time span of 18 h. Only the colonies able to propagate in this liquid medium were also tested for the capability to metabolise filter paper (FP), using the screening medium FP-SM (Additional file 1: Table S1). To test if modifications of the FP texture could occur only as consequence of the stirring and high temperature applied, a control flask containing FP-SM without cells was incubated for the same time span at 60 °C.

### Identification, physiological characterisation and phylogenetic analysis of a new cellulolytic *Bacillus coagulans* strain

The best-performing isolate was identified as a strain of *B. coagulans* by 16S rRNA-encoding gene sequencing and cellular fatty acid analysis performed by a certified laboratory (DSMZ), and it is hereafter referred to as *B. coagulans* MA-13. Furthermore, the 16S sequence (GenBank accession number; MF373392) was used to search the GenBank database by means of BLASTn [34]. 16S rRNA sequences of several representative *B. coagulans* strains were retrieved, aligned with T-COFFEE and a phylogenetic tree (1000 bootstrap replicates) was constructed with the MEGA6 software using the Maximum Likelihood method based on the Tamura–Nei model [35]. The 16S sequence from *Bacillus circulans* strain 5S5 was used as an outgroup to root the tree.

To further characterise this strain, standard physiological and biochemical tests were performed including: (1) motility assay; (2) Gram staining; (3) the Voges–Proskauer (VP) test; (4) catalase, oxidase, lecithinase, phenylalanine deaminase and arginine dihydrolase activity assays; (5) nitrate reduction test; (6) indole production; (7) citrate and propionate utilisation as well as (8) organic acids and gas production from glucose. Additionally, the strain was tested for its ability to hydrolyse different biopolymers, such as casein, gelatine, starch and Tween 80.

### Determination of the optimal temperature of growth

To assess the effect of the temperature on the growth of *B. coagulans* MA-13, a pre-culture was cultivated using LB medium in a 250-ml Erlenmeyer flask at 60 °C and shaking speed of 180 rpm for 4 h (up to an OD_600_ value of 1.5). This latter was used to seed 50-ml aliquots of CMC-SM medium (Additional file 1: Table S1) at an initial OD_600_ of 0.08. These cultures were then incubated at 37, 50, 55 and 60 °C, in 250-ml Erlenmeyer flasks with a long neck (to avoid evaporation) in a rotary shaker at 180 rpm. Optical density was measured regularly over a time span of 15 h. The steepest part of the ln (OD_600_) curves, assessed by at least four measurement points, was used to calculate both the maximum specific growth rates (*µ*_max_) and the coefficient of determination (*R* ^2^). Three independent replicates of the experiment were performed using different precultures.

### Determination of endoglucanase activity in the culture supernatant and soluble proteins detection

The strain *B. coagulans* MA-13 was cultivated in liquid CMC-SM (Additional file 1: Table S1) at 55 °C up to the stationary phase (about 15 h) and harvested by centrifugation at 3000*×g* for 15 min. Proteins secreted by *B. coagulans* MA-13 were precipitated by gradually adding (NH_4_)_2_SO_4_ into the cell-free culture supernatant, reaching a final concentration of 90% (w/v). After stirring for 1 h, the supernatant was centrifuged at 5000×*g* for 15 min (4 °C) and the obtained protein pellet was resuspended and dialysed in 50 mM Tris–HCl pH 7.0 (AppliChem). Protein concentration was determined according to Bradford assay [36], using serial dilutions of bovine serum albumin as standards. A preliminary detection of endoglucanase enzymes in the cell-free culture supernatant was performed by means of the CMC-hydrolysis spot assay. The concentrated and dialysed culture supernatant was spotted (5 μg of total proteins) on agar plates containing 1% (w/v) CMC and incubated at 55 °C for 30 min. In parallel, a positive control of degradation was performed, by spotting the same amount of a commercially available enzyme solution (Cellulases from *Trichoderma reesei* ATCC 26921; Sigma-Aldrich). After staining with 0.1% (w/v) Congo Red (Sigma-Aldrich), degradation haloes were revealed by washing the plate surface with 1 M NaCl (AppliChem) and fixing with 5% (v/v) acetic acid (Carlo Erba).

To confirm the presence of soluble proteins, the concentrated and dialysed cell-free culture supernatant was subjected to a two-step purification procedure through cation-exchange and size-exclusion chromatography. To achieve this aim, the sample was loaded onto a 1-ml cation-exchange Resource S column (GE Healthcare) connected to a fast-performance liquid chromatography system (ÄKTA Explorer; GE Healthcare). The column was equilibrated with 50 mM Tris–HCl pH 7.0 and the elution was performed with a linear gradient of NaCl from 0 to 800 mM. Chromatography fractions were tested for endo-1,4-β-glucanase activity, using Azo-CMC as substrate (Megazyme) and following the supplier’s instructions. All positive fractions were pooled together in the same tube, dialysed against 50 mM Tris–HCl and 200 mM NaCl, pH 7.0 and concentrated to a final volume of approximately 0.2 ml, using a Centricon^®^ membrane with a 3-kDa cut-off (Millipore). The concentrated sample was subjected to size-exclusion chromatography using a Superdex PC75 column (3.2 by 30 cm; GE Healthcare) equilibrated with 50 mM Tris–HCl pH 7.0 and eluted at a flow rate of 0.04 ml/min. Protein fractions displaying Azo-CMCase activity were pooled and analysed by 12% SDS-PAGE [37].

### Time course of the enzyme secretion into the culture supernatant

To set up a cultivation medium for the induction of enzymes secretion, *B. coagulans* MA-13 was grown at 55 °C in media containing: (1) glycine-buffered Brock’s basal salt solution [38–41]; (2) 0.2% (w/v) nitrogen source [either (NH_4_)_2_HPO_4_, tryptone or yeast extract] and (3) 0.5% (w/v) CMC. Among the nitrogen sources, tryptone was found to better induce the secretion of enzymes and the corresponding medium was named carboxymethyl-cellulose induction medium (CMC-IM, Additional file 1: Table S1). CMC-IM was used to investigate if *B. coagulans* MA-13 was able to secrete endoglucanases at a lower temperature, by performing a time course analysis at 37 °C. In particular, 50 ml of CMC-SM was seeded with a glycerol stock of *B. coagulans* MA-13 and incubated under constant shaking (180 rpm) at 55 °C for 4 h (up to 1.5 OD_600_). This pre-culture was then used to inoculate a 50-ml aliquot of CMC-IM at an initial OD_600_ of 0.08. This second phase of cultivation was carried out at 37 °C and 180 rpm. In parallel, LB medium was used as a negative control of enzyme secretion. Aliquots were regularly withdrawn to measure the optical density as well as to obtain cell-free supernatants though centrifugation at 4000×*g* for 10 min. Endo-1,4-β-glucanase activity of the cell-free supernatants was assayed at three different temperatures (37, 50 and 60 °C), using Azo-CMC as substrate (Megazyme) and following supplier’s instructions. Three independent replicates of this experiment were carried out using different precultures.

### Comparison of *Bacillus coagulans* MA-13 growth on different sugars and hydrolysate concentrations

The growth of *B. coagulans* MA-13 was monitored in microscale conditions (145 µl) at 55 °C and pH 5.5, using the Bioscreen C MBR equipment (Oy Growth Curves Ab Ltd). Although the bacterium shows an optimal growth at pH 6.0, it is also able to propagate well at pH 5.5 (Additional file 1: Figure S1). Therefore, subsequent characterisations were carried out at pH 5.5, since acidic conditions are commonly used for the microbial production of LA at industrial scale. To test lignocellulose-derived sugars utilisation, media supplemented with trace metals and vitamins [42] and containing different sugars were used (Additional file 1: Table S1).

The strain robustness was tested by cultivations in the liquid fraction (hydrolysate) of a wheat straw slurry, pre-treated by sulfuric acid-catalysed hydrolysis and steam explosion as described elsewhere [43] and containing inhibitors such as 3.8 g/l of acetic acid, 4.0 g/l of furfural and 1.4 g/l of 5-hydroxymethylfurfural (HMF); concentrations were determined by HPLC (see below). The pH of the hydrolysate was adjusted to 5.5 using concentrated NaOH, before being mixed with the above-mentioned medium and molasses as a source of sucrose [43] to achieve concentrations of hydrolysate ranging from 10 to 80% (v/v). Prior to microscale cultivations, *B. coagulans* MA-13 was grown into the stationary phase in LB medium at 55 °C and shaking speed of 180 rpm. To remove nutrients leftover from the LB medium, the culture was centrifuged at 3000×*g* for 10 min at room temperature and the cell pellet was resuspended in 1% (w/v) NaCl to an OD_600_ value of about 3.0. This suspension was used to seed each well of the Bioscreen C plate at an initial OD_600_ of about 0.1. To avoid sample evaporation, the multi-well plate was sealed with a transparent adhesive tape. Then, it was placed into a Bioscreen C MBR apparatus set at high amplitude and fast speed. At 20-min intervals, mixing was stopped for 5 s prior to each optical measurement (OD_600_).

The recorded cell density values were converted to equivalent OD_600_ using the following Eq. (1):

1 $$ {\text{OD}}_{\text{observed}} = \frac{{{\text{OD}}_{\text{bioscreen}} }}{{{\text{Pathlength}}\,\left( {\text{cm}} \right) \times 1.32}}. $$

The non-linear correlation between optical density and cell density was corrected using Eq. (2) [40]:

2 $$ {\text{OD}}_{\text{corrected}} = {\text{OD}}_{\text{observed}} + 0.449 \times {\text{OD}}_{\text{observed}}^{2} + 0.191 \times {\text{OD}}_{\text{observed}}^{3}. $$

The steepest part of the ln (OD_corrected_) curve, using 12 measurement points, was used to calculate both the maximum specific growth rate (*µ*_max_) and the coefficient of determination (*R* ^2^). For each experimental condition, a minimum of nine technical replicates were included in the plate. Moreover, two independent replicates were carried out using different starting seed cultures (for a total of 18 replicates).

### Fermentation

Fermentations were performed in 3.6 l bioreactor vessels (INFORS HT), autoclaved at 121 °C for 20 min before usage. Inoculum cultures were grown in 500 ml of LB medium in a 2.0 l flask at 55 °C and shaking speed of 180 rpm for 3–4 h (up to an OD_600_ value of about 1.0-1.3). Culture aliquots (about 100 ml) were centrifuged at 4000×*g* for 10 min and the obtained bacterial pellets were resuspended using 20 ml of sterile medium collected from each bioreactor vessel. Fermentations were initiated by inoculating the cell suspension into the bioreactors through a sterile rubber septum at an initial OD_600_ of about 0.1. The composition of the batch fermentation media is reported in the Additional file 1: Table S1. Each batch fermentation was carried out in 1 l medium at 55 °C at a stirring speed of 500 rpm, until complete sucrose consumption. Nitrogen was sparged at 1 VVM (volume of gas/volume of medium/minute), and pH was kept at 5.5 by adding 3 M NaOH. Antifoam 204 (Sigma-Aldrich) was added as required. Optical density was spectrophotometrically measured at 600 nm and the steepest part of the ln(OD_600_) curves, estimated by linear regression over five points, was used to calculate both the maximum specific growth rates (*µ*_max_) and the coefficient of determination (*R* ^2^). The concentrations of sucrose and fermentation products (lactate, acetate and acetoin) were determined by HPLC analysis of the culture supernatant, as described below.

### HPLC analysis

Culture samples were centrifuged at 4000×*g* for 5 min and supernatants were filtered using 0.2 μm sterile filters (VWR^®^). High-performance liquid chromatography (HPLC) and quantification of sucrose, lactate, acetate and acetoin was performed by means of an UltiMate 3000 HPLC system equipped with a variable wavelength absorbance detector set at 210 nm (Dionex) and an IR-101 refractive index detector (Shodex), using a Rezex ROA column (Phenomenex). The analysis was carried out at a column temperature of 80 °C, using 5 mM H_2_SO_4_ as the mobile phase at a flow rate of 0.8 ml/min throughout the analysis.

## Results

### Isolation, identification and characterisation of a new *Bacillus coagulans* strain

**Fig. 1:**
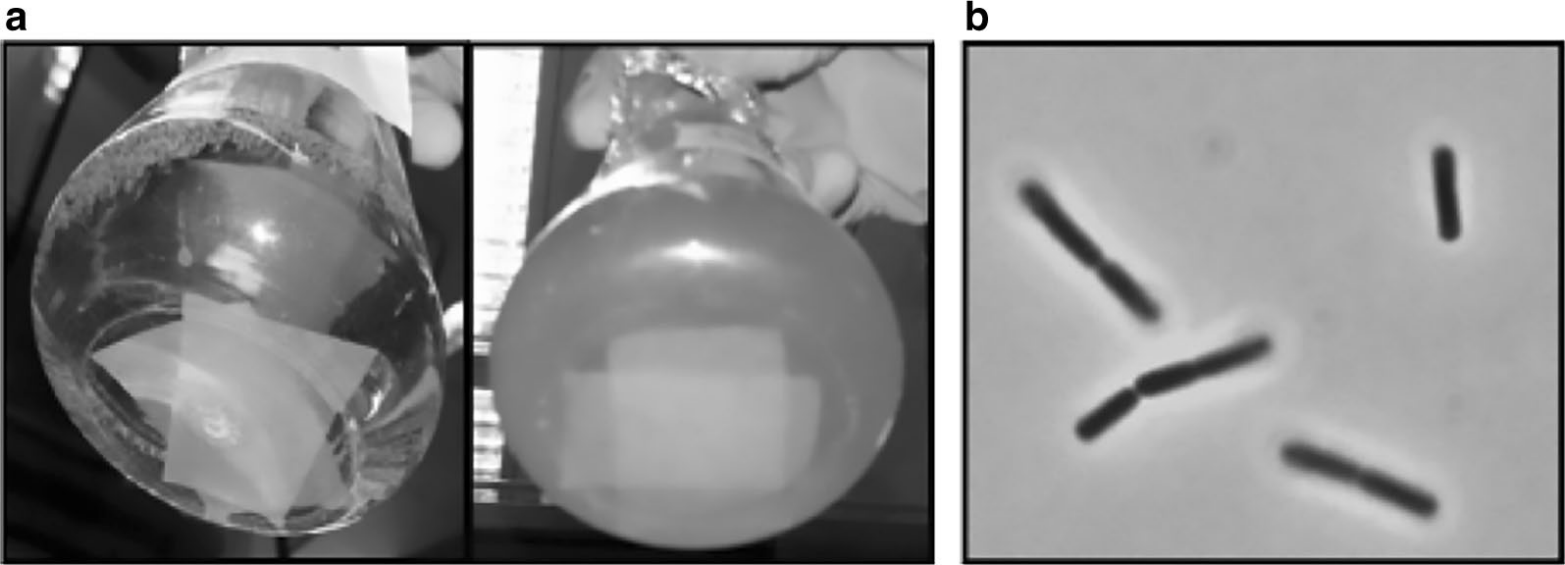
Isolation of the cellulolytic strain MA-13. **a** *Bacillus coagulans* MA-13 culture after overnight growth in FP-SM (*right side*) and negative control without cells (*left side*). **b** Light microscope observation of *B. coagulans* MA-13 cells when cultivated in CMC-SM (Additional file 1: Table S1)

**Fig. 2:**
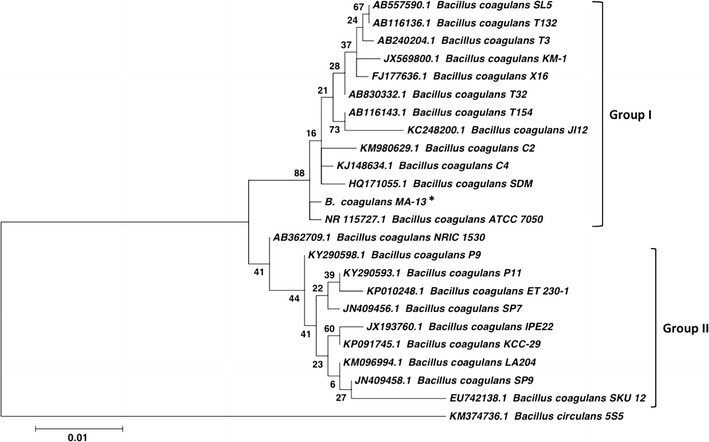
Phylogenetic relationship between MA-13 and other *B. coagulans* strains based on 16S rRNA sequences. The evolutionary history was inferred by using the Maximum Likelihood method based on the Tamura–Nei model [35]. The tree with the highest log likelihood (−1664.1025) is shown. The percentage of trees in which the associated taxa clustered together is shown next to the branches. Initial tree(s) for the heuristic search were obtained by applying the Neighbour-Joining method to a matrix of pairwise distances estimated using the Maximum Composite Likelihood approach. The tree is drawn to scale, with branch lengths measured in the number of substitutions per site (0.01). The analysis involved 24 nucleotide sequences. All positions containing gaps and missing data were eliminated. There was a total of 745 positions in the final dataset. Evolutionary analyses were conducted in MEGA6, using the 16S sequence from *Bacillus circulans* 5S5 as an outgroup to root the tree. For all strains, the GenBank accession number is reported

Hundreds of colonies from CMC-SM plates, seeded with an agricultural waste suspension, were tested for the ability to grow in liquid medium. In this condition, only five isolates could propagate in CMC-SM, thus confirming their ability to metabolise the polymeric carboxymethyl-cellulose substrate. These cellulolytic isolates were cultivated also in liquid FP-SM medium (Fig. 1a). Among these, the one named MA-13 achieved the highest optical density (OD_600_) in a time span of 24 h, when cultivated in both CMC-SM and FP-SM media. Subsequently, MA-13 was identified as a strain of *B. coagulans* through 16S rRNA-encoding gene sequencing and cellular fatty acids profiling (DSMZ). The phylogenetic tree based on 16S rRNA sequences showed two main branches (Group I and Group II) that included a similar number of *B. coagulans* strains. Although *B. coagulans* MA-13 clustered in Group I, it appears more phylogenetically distant from the other members of the same branch, similarly to the strain ATCC7050 (Fig. 2).

**Fig. 3:**
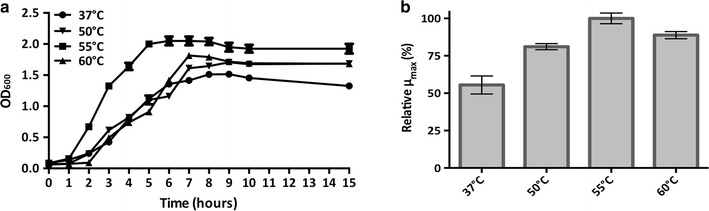
Assessment of the optimal temperature of growth. **a** Average growth curves (*n* = 3) at different temperatures in CMC-SM (Additional file 1: Table S1). **b** *Bar plots* of the maximum specific growth rates (*μ*_max_) expressed as percentage (%) relative to the optimal condition (55 °C). *Error bars* show the deviation between triplicate experiments

*Bacillus coagulans* MA-13 turned out to be a facultative anaerobic, spore-forming, Gram-positive, motile, rod-shaped bacterium (Fig. 1b; Table 1), which hydrolyses biopolymers such as gelatine, starch and casein (Table 1). Moreover, like other *Bacillus* species, MA-13 is capable of denitrification, the respiratory reduction of nitrate and nitrite to N_2_O (Table 1). To assess its optimal temperature of growth, *B. coagulans* MA-13 was cultivated in liquid CMC-SM at different temperatures (Fig. 3a) and it showed the highest relative growth rate at 55 °C (Fig. 3b).

**Table 1:** Physiological and biochemical properties of *Bacillus coagulans* MA-13

Features	Reaction	Carried out
Gram staining	+	by DSMZ
Voges–Proskauer reaction	−	by DSMZ
Gas from glucose	−	by DSMZ
Spores	+	by DSMZ
Motility	+	by DSMZ
Denitrification		
NO_3_ → NO_2_	+	by DSMZ
Indol reaction	−	by DSMZ
Oxidase	−	by DSMZ
Growth		
30 °C	+	by DSMZ
37 °C	+	in this work
40 °C	+	by DSMZ
50 °C	+	by DSMZ and in this work
55 °C	+	in this work
60 °C	+	in this work
Growth in medium pH 4.5	+	in this work
pH 5.0	+	in this work
pH5.5	+	in this work
pH 5.7	+	by DSMZ
pH6.0	+	in this work
pH7.0	+	in this work
NaCl 2%	+	by DSMZ
NaCl 5%	−	by DSMZ
Lysozyme-broth	+	by DSMZ
Aerobic	+	in this work
Anaerobic	+	by DSMZ and in this work
Acid fermentation of		
d-glucose	+	by DSMZ and in this work
d-mannose	+	in this work
d-galactose	−	in this work
d-arabinose	−	by DSMZ and in this work
l-arabinose	−	in this work
d-xylose	−	by DSMZ and in this work
d-mannitol	−	by DSMZ
d-fructose	+	by DSMZ and in this work
d-melibiose	+	in this work
d-cellobiose	+	in this work
Lactose	+	in this work
Hydrolysis of		
Casein	+	by DSMZ
Gelatine	+	by DSMZ
Starch	+	by DSMZ
Carboxymethyl-cellulose	+	in this work
Filter paper	+	in this work
Tween 80	−	by DSMZ
Enzymes production		
Phenylalanine deaminase	−	by DSMZ
Arginine dihydrolase	−	by DSMZ
Lecithinase	−	by DSMZ
Catalase	+	by DSMZ
Endo-1,4-β-glucanase	+	in this work

+, positive reaction; −, negative reaction

### *Bacillus coagulans* MA-13 secretes cellulases into the culture supernatant

**Fig. 4:**
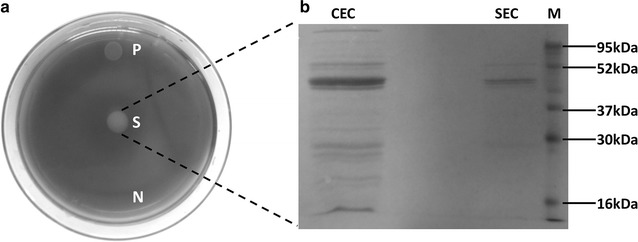
Detection of secreted endoglucanase enzymes. **a** CMC-hydrolysis spot assay. Positive control (P): cellulase from *Trichoderma reesei* ATCC 26921 (5 μg of total proteins; determined by Bradford assay). Negative control (N): 90% (w/v) (NH_4_)_2_SO_4_ solution. Concentrated supernatant (S): *B. coagulans* cell-free supernatant concentrated by adding 90% (w/v) (NH_4_)_2_SO_4_ (5 μg of total proteins; determined by Bradford assay). **b** SDS-PAGE of Azo-CMCase positive fractions obtained by cation-exchange (CEC) and size-exclusion chromatography (SEC). Molecular mass markers (M)

The ability of *B. coagulans* MA-13 to utilise both CMC and FP as carbon sources suggested that this bacterium might produce cellulolytic enzymes for the hydrolysis of these substrates. To understand whether these enzymes were secreted into the medium, the cellulolytic activity of the cell-free supernatant was assayed. To this end, *B. coagulans* MA-13 was cultivated at 55 °C in liquid CMC-SM for 15 h, and the concentrated cell-free supernatant was spotted on CMC-agar plates. The appearance of a degradation halo after Congo Red staining proved the presence of cellulases in the culture supernatant (Fig. 4a). To further confirm the secretion of soluble enzymes, the concentrated cell-free supernatant was dialysed and partially purified through cation-exchange and size-exclusion chromatography. All the fractions were assayed for endo-1,4-β-glucanase activity and the positive ones were pooled, concentrated and analysed on SDS-PAGE (Fig. 4b). Although several protein bands were visible on the gel, the predominant one migrated in the range of 40–50 kDa, which is consistent with the molecular weight of other bacterial cellulases [29, 45, 46].

**Fig. 5:**
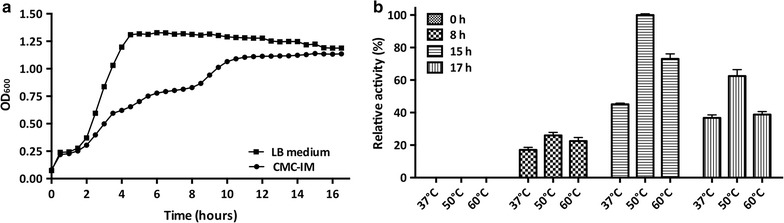
Time course secretion of endoglucanase enzymes. **a** Average growth curves (*n* = 3) at 37 °C in CMC-IM (Additional file 1: Table S1) and LB medium (negative control of secretion). **b** *Bar plots* of Azo-CMCase activity tested at three different temperatures. Values are expressed as percentage (%) relative to the maximum value (i.e. at 15 h at 50 °C). *Error bars* show the deviation between triplicate experiments

To assess the production of enzymes at a lower temperature, *B. coagulans* MA-13 was cultivated at 37 °C in both CMC-IM and in LB medium; this latter as a negative control of secretion (Fig. 5a). As expected, the LB medium culture supernatant did not show any endo-1,4-β-glucanase activity when tested by Azo-CMC assay (data not shown). When the bacterium was grown in CMC-IM, no Azo-CMCase activity was detected during the first 7 h of cultivation (data not shown). Nevertheless, after a short stationary phase (Fig. 5a, 6–8 h), endo-1,4-β-glucanase activity was observed in the culture supernatant (Fig. 5b, 8 h). The enzymes secretion was accompanied by the onset of an additional short exponential growth phase (Fig. 5a, 8–10 h) and the highest enzymatic activity was detected after 15 h of cultivation (Fig. 5b). Furthermore, the enzymes in the supernatant were found to be active at three different temperatures (i.e. 37, 50 and 60 °C), although they performed better at 50 °C (Fig. 5b). Interestingly, when *B. coagulans* MA-13 was cultivated at its optimal temperature of growth (55 °C), the secretion of enzymes into the culture supernatant was comparable to that of cells cultivated at 37 °C (data not shown).

### *Bacillus coagulans* MA-13 is able to metabolise lignocellulose-derived sugars

**Fig. 6:**
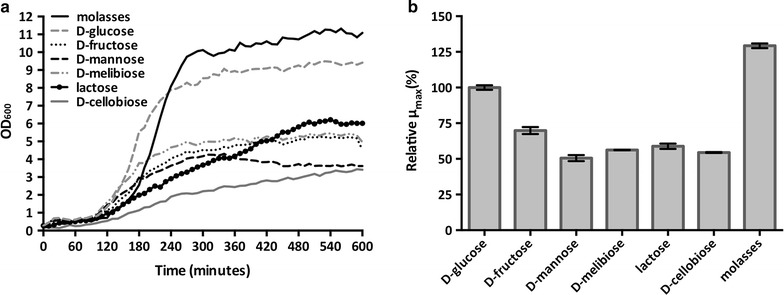
Cultivation using different lignocellulose-derived sugars. **a** Average growth curves in Bioscreen media containing different saccharides (Additional file 1: Table S1). **b** *Bar plots* of the maximum specific growth rates (*μ*_max_) expressed as percentage (%) relative to the *μ*_max_ in presence of glucose. *Error bars* show the deviation between duplicate experiments

Several Gram-positive bacteria are able to ferment sugars to a wide palette of organic acids [47]; among these, *B. coagulans* is a well-known LA producer [9, 24, 48, 49]. For this reason, the ability of the strain MA-13 to utilise different sugars and to produce LA was tested. In particular, this bacterium was cultivated on several lignocellulose-derived monosaccharides (d-glucose, d-mannose, d-fructose, l-arabinose, d-xylose and d-galactose) and disaccharides (d-melibiose, d-cellobiose and lactose). Except for d- and l-arabinose, d-xylose and d-galactose, *B. coagulans* MA-13 was found to grow using all the sugars tested (Fig. 6a). Since glucose was the preferred carbon source, yielding the highest maximum specific growth rate (*μ*_max_), it was used as reference (100%) to calculate the relative *μ*_max_ for the other saccharides. Compared to d-glucose, the *μ*_max_ for d-fructose was about 30% lower, whereas for d-mannose, d-melibiose, d-cellobiose and lactose it was reduced by about 50% (Fig. 6b). *B. coagulans* MA-13 was also cultivated using the commonly used and relatively inexpensive carbon source, molasses, which contains primarily sucrose [43]. Interestingly, in this case, the relative *μ*_max_ was even higher than that showed for d-glucose (Fig. 6b).

To assess the presence of organic compounds typically produced either aerobically (acetate and acetoin) or anaerobically (lactate), an end-point HPLC analysis was carried out for all the tested conditions. However at rather low concentration these compounds were produced from all the sugars analysed (Additional file 1: Table S2), except for d-cellobiose that yielded only acetate and acetoin. The lack of control over important parameters such as aeration, pH and proper mixing under microscale conditions can explain the low concentration of the organic compounds. On the other hand, their concomitant presence can be accounted to the microaerophilic environment of the plate, which may allow both aerobic and anaerobic metabolism.

### The new isolate is tolerant to a steam-exploded wheat straw hydrolysate

**Fig. 7:**
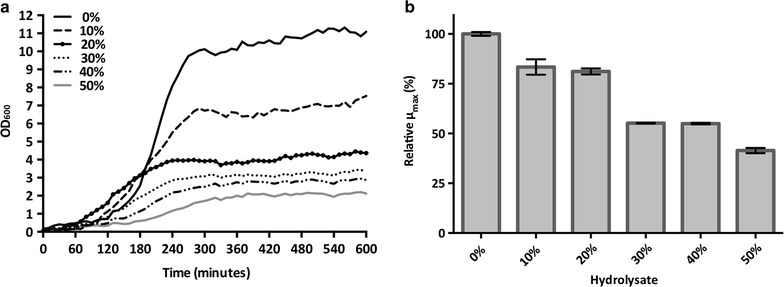
Effect of the steam-exploded wheat straw hydrolysate on the growth of *B. coagulans* MA-13. **a** Average growth curves in Bioscreen media containing different concentrations of hydrolysate (Additional file 1: Table S1). **b** Maximum specific growth rates (*μ*_max_) expressed as percentage (%) relative to the rate in the absence of hydrolysate (0%). *Error bars* show the deviation between duplicate experiments

The microscale cultivation at increasing concentration of steam-exploded wheat straw hydrolysate [43] was performed to test the inhibitory effect on the bacterium propagation (Fig. 7a) and on the production of LA (Additional file 1: Table S3). The relative *µ*_max_ was reduced compared to the control, but in a non-linear fashion (Fig. 7b). Only a slight reduction of the relative *µ*_max_ to about 80% was observed at low hydrolysate concentrations (10 and 20%, Fig. 7b). However, at both 30 and 40% concentrations of hydrolysate, a drop in the *µ*_max_ by about 50% was detected (Fig. 7b). Only a further 10% reduction of the *µ*_max_ was observed at 50% hydrolysate (Fig. 7b), whilst the growth was completely inhibited at 60% hydrolysate (data not shown). Moreover, the end-point HPLC analysis showed that the production of lactate was negatively affected by the presence of hydrolysate (Additional file 1: Table S3).

### *Bacillus coagulans* MA-13 is less inhibited by the hydrolysate in a controlled batch fermentation set-up

**Fig. 8:**
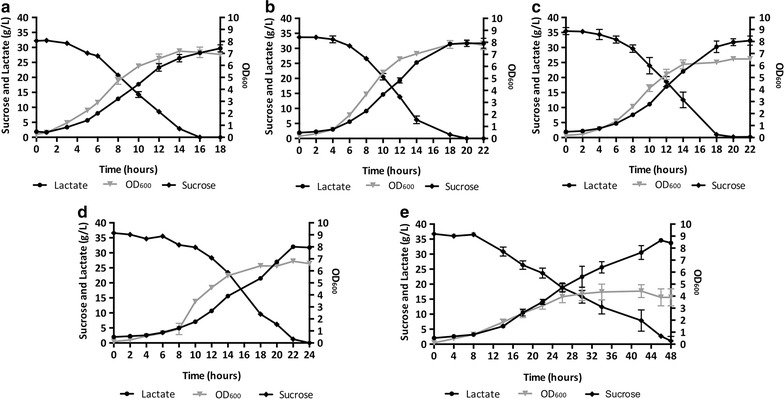
*B. coagulans* anaerobic batch fermentations. Growth and fermentation profiles in hydrolysate-free (**a**) as well as in hydrolysate-containing media: 30% (**b**), 50% (**c**), 70% (**d**) and 95% (**e**). For the composition of fermentation media see Additional file 1: Table S1. *Error bars* show the deviation between duplicate experiments

Although microscale cultivation using Bioscreen C allows assessing several conditions simultaneously, this system suffers from the lack of regulation of crucial parameters such as aeration and pH control. For this reason, both bacterial growth and lactic acid production were studied in controlled bioreactor conditions (Fig. 8). When anaerobically cultivated in a hydrolysate-free medium (Fig. 8a), *B. coagulans* MA-13 consumed all the sucrose in 16 hours, with a yield of 0.92 *g*_lactate_/*g*_sucrose_. The average and maximum volumetric productivities of LA were 1.40 and 2.55 g/l/h, respectively, whereas the average and maximum specific productivities were 0.28 and 0.50 g/l/h/OD, respectively (Table 2).

**Table 2:** Effect of the hydrolysate on the growth and lactic acid production of *B. coagulans* MA-13

Batch name	*μ*_max_ (h^−1^)	Relative *μ*_max_ (%)	Final LA concentration (g/l)	LA maximum volumetric productivity (g/l/h)	LA maximum specific productivity (g/l/h/OD)	LA average volumetric productivity (g/l/h)	LA average specific productivity (g/l/h/OD)	LA yield (g/g)	Fermentation time (h)
Hydrolysate-free	0.44	100	29.70	2.55	0.50	1.40	0.28	0.92	18
30% hydrolysate	0.37	84	32.05	2.70	0.42	1.25	0.23	0.95	22
50% hydrolysate	0.35	78	32.30	2.40	0.54	1.38	0.30	0.91	22
70% hydrolysate	0.30	67	31.77	1.81	0.63	1.24	0.30	0.91	24
95% hydrolysate	0.26	58	33.71	1.18	0.99	0.59	0.38	0.92	48

Unlike what was observed in microscale cultivations (Fig. 7b), the *µ*_max_ was only slightly reduced in 30 and 50% hydrolysate media (Table 2) when grown under controlled bioreactor conditions (Fig. 8b–d). Interestingly, the microbial growth was never completely inhibited, but was only reduced by about 40% even at the highest hydrolysate concentration (Table 2). On the other hand, the increase in the hydrolysate concentration in the fermentation medium was accompanied by decreased average and maximum volumetric LA productivities (Table 2). Although this had an effect on the process time, the variation of the LA yield appeared to be negligible. Interestingly, both the average and maximum biomass-specific LA productivities increased proportionally to the hydrolysate concentration in the medium (Table 2).

## Discussion

Lignocellulosic biomasses, such as municipal, agricultural and forestry residues, are attractive renewable sources for the eco-friendly production of biofuels and chemicals. Nevertheless, to be sustainably used they need to be processed in order to release monomeric sugars that can be fermented or otherwise converted to value-added products [2]. In this context, microorganisms represent both reservoirs of hydrolytic enzymes [21, 22] as well as potential cell factories for several bioconversion processes [47]. In nature, decomposition of lignocellulosic materials requires complex processes, with the participation of several lignocellulolytic microorganisms. The best biological niches for the isolation of such microorganisms are those where cellulose-containing substrates are present [50]. Therefore, thermophilic and cellulolytic microorganisms were isolated from an agricultural residue, and were screened for their ability to grow at high temperature using FP and CMC as carbon sources (Figs. 1a, 3a). The isolate *B. coagulans* MA-13 stood out for its rapid utilisation of these complex substrates, and when tested at different temperatures in a CMC-containing medium, 55 °C was found to be its optimal temperature of growth (Fig. 3).

The ability of several *Bacillus* species to secrete a wide range of hydrolytic enzymes [51, 52], together with the evidence that the strain MA-13 metabolises both CMC and FP, suggests that this bacterium might secrete lignocellulolytic enzymes. Moreover, thermophilic microorganisms are of huge interest since they are endowed with more stable enzymes [23]. For these reasons, the presence of soluble cellulases in the cell-free culture supernatant of *B. coagulans* MA-13 was preliminary tested by CMC-hydrolysis spot assay. Interestingly, the resulting degradation halo was comparable to that generated by a commercially available enzyme cocktail (Fig. 4a). Besides being tested by spot assay, the occurrence of cellulolytic enzymes in the bacterial supernatant was confirmed by partial purification (Fig. 4b) and Azo-CMC assay. To test if *B. coagulans* MA-13 could be a source of endoglucanases, even when cultivated at lower temperature, the time course of secretion was investigated at 37 °C. In the presence of CMC this bacterium showed a biphasic growth curve, with enzymes secretion occurring only after a first short stationary phase (Fig. 5a, 6–8 h), hinting that the shortage of some nutrients in the medium might trigger the production of cellulases. It is worth noting that, despite the low temperature of cultivation (37 °C), the secreted enzymes performed better at 50 °C (Fig. 5b), which overlaps the optimal operational temperature of commercial hydrolytic enzyme cocktails (50–55 °C) used for the saccharification of lignocellulosic biomasses [24]. Unlike the *B. coagulans* strain PFH N7 that showed a decreased enzyme secretion at temperatures higher than 37 °C [53], the secretion of endo-1,4-β-glucanase enzymes by the strain MA-13 appears not to be influenced by the temperature of growth. Indeed, the enzyme activity in the supernatant was comparable when this bacterium was cultured at both 37 and 55 °C (data not shown).

Many strains of *B. coagulans* are generally regarded as safe (i.e. GRAS) by the U.S. Federal Drug Administration (FDA) [54–56]. Therefore, besides being used as a chemical for PLA manufacturing, the LA produced by this bacterium might be potentially used as an additive in pharmaceutical and cosmetic applications as well as in food industries [7]. Moreover, *B. coagulans* is one of the model organisms used for the production of LA from lignocellulose biomasses [8, 23, 45, 46]. For this reason, the strain M-13 was tested for the consumption and fermentation of different cellulose- and hemicellulose-derived sugars. Since *B. coagulans* MA-13 was selected for its capability to degrade cellulose, it was not surprising to observe that, besides d-mannose, it could not metabolise other hemicellulose-derived sugars (i.e. d- andl-arabinose, d-xylose and d-galactose). On the other hand, the utilisation of cellulose-derived sugars was expected (Fig. 6; d-glucose and d-cellobiose). The highest relative µ_max_ was observed when molasses was provided as carbon source (Fig. 6b). This is of particular interest because molasses is a renewable, easily available and relatively inexpensive source of sugars that is often used at industrial scale [57, 58]. The improved growth in molasses has already been observed for other *Bacillus* species [59, 60] and might be related to the presence of growth promoters such as vitamins, organic compounds and minerals.

Another desirable feature of microorganisms employed for the production of eco-friendly fuels and chemicals is the tolerance towards inhibitors released during the pre-treatment of the lignocellulosic biomass [14–16]. To this end, the robustness of *B. coagulans* MA-13 was investigated by cultivation in the liquid fraction (hydrolysate) of an acid and steam-exploded wheat straw slurry [43]. Cell growth in media containing 10 and 20% hydrolysate was only slightly affected compared to the growth in hydrolysate-free medium, but the relative *µ*_max_ dropped significantly at 30–50% of hydrolysate (Fig. 7). Cell growth was completely inhibited at concentrations above 50%. Interestingly, these data show that *B. coagulans* MA-13 is indeed a robust bacterium able to withstand hydrolysate concentrations higher than those tolerated in batch cultivations by *Saccharomyces cerevisiae* using the same hydrolysate [43], i.e. a model organism widely used for lignocellulosic ethanol production. Moreover, *B. coagulans* MA-13 appears to better tolerate furfural than what is shown for the thermophilic *Bacillus* spp. IFA 119. Indeed, whereas in microscale cultivation MA-13 showed a 60% reduction of its relative *µ*_max_ in the medium containing 50% hydrolysate (about 2.0 g/l of furfural), the growth of the strain IFA 119 was inhibited by 96% when cultivated in a medium containing only 0.8 g/l of furfural [61]. On the other hand, the growth of *B. coagulans* MXL-9 was not inhibited by furfural up to 2.5 g/l [62], although its behaviour was never tested in the presence of a complex mixture of inhibitors, such as the acid and steam-exploded wheat straw hydrolysate used in this work.

To study the behaviour of *B. coagulans* MA-13 in a more controlled experimental set-up, batch fermentations were carried out in high-performance bioreactors using hydrolysate-free medium as well as at concentrations of hydrolysate exceeding those tolerated in the microscale cultivations. Under strict anaerobic conditions the bacterium produced only LA, thus confirming its homo-fermentative nature. Moreover, the better mixing, together with stringent anaerobic condition and pH control throughout the cultivation in bioreactors, alleviated the detrimental effect of the hydrolysate inhibitors on the bacterial growth. In fact, compared to what was observed in a hydrolysate-free medium, in the presence of 95% hydrolysate the bacterial growth was reduced only by about 50% (Fig. 8; Table 2). This indicates that *B. coagulans* MA-13 is more robust than *B. coagulans* IPE22 (Fig. 2, Group II), the growth of which was reduced by about 75% when cultivated in a medium containing only furfural (3.0 g/l) as inhibitory compound [49]. Therefore, MA-13 might be an even more suitable strain for lignocellulosic LA production (Fig. 8; Table 2) than previously indicated by the microscale cultivation data (Fig. 7). Concerning the fermentation performance, it is noteworthy that the LA yield was similar in all the tested conditions. In contrast, the volumetric productivities were reduced in the hydrolysate-containing media (Table 2). These results show that the inhibitory compounds present in the hydrolysate [43] had no effect on the final lactate yield, whilst they affected the bacterial growth and, in turn, the volumetric LA productivities (Table 2). These data suggest that the fermentation rate is linked to the bacterial growth. Indeed, the time to complete the fermentation was extended from 18 h in the hydrolysate-free medium to 48 h in the 95%-hydrolysate-containing medium (Table 2). However, the specific LA productivities increased with the hydrolysate concentration (Table 2). This can be explained by a higher cellular requirement of energy to be used in stress responses and for detoxification of inhibitors at increased hydrolysate concentrations. Since sugar fermentation is the only way to produce energy (i.e. reducing power and ATP) in anaerobic conditions, this will in turn stimulate the production of LA.

Interestingly, once again, *B. coagulans* MA-13 was shown to be more robust than other strains such as *B. coagulans* JI12 (Fig. 2, Group I) and NL01; in fact, these latter showed a fermentation time of 48 h at lower concentration of furfural (1.4 and 0.18 g/l) and HMF (0.3 and 0.37 g/l), respectively [63, 64]. Altogether the features displayed by *B. coagulans* MA-13, i.e. (1) the secretion of thermophilic cellulases; (2) the utilisation of lignocellulose-derived sugars and (3) the tolerance towards lignocellulose hydrolysate inhibitors, make this bacterium an appealing candidate for the production of LA from lignocellulosic biomasses. Nonetheless, given the presence of 20–40% of hemicellulose in lignocellulosic biomass, further development of the MA-13 strain is required to provide this strain with the ability to ferment hemicellulose-derived sugars (e.g. xylose). Indeed, this is a desirable trait for LA production strains, since it allows exploiting the full polysaccharide composition of the biomass and, in turn, to increase the final LA titre. Moreover, the future design of a consolidated bioprocess, via genetic and evolutionary engineering of MA-13, can be envisaged since genetic accessibility has already been reported for *B. coagulans* [33].

## Conclusions

In the present work, a novel thermophilic and cellulolytic strain of *B. coagulans* MA-13 was isolated from canned beans manufacturing residues for its ability to metabolise both CMC and FP. MA-13 is a facultative anaerobic bacterium showing an optimal temperature of growth of 55 °C, which is able to secrete soluble endo-1,4-β-glucanase enzymes into the culture supernatant, even when cultivated at a lower temperature (37 °C). Although the secreted enzymes are active in a wide range of temperature (37–60 °C), they perform better at 50 °C. MA-13 was also characterised for the production of lactic acid in media containing a hydrolysate derived from the pre-treatment of wheat straw by acid-catalysed hydrolysis and steam explosion. Whereas the fermentation process was completed within 18 h in a hydrolysate-free medium, the process time was extended to 48 h at the highest hydrolysate concentration tested (95% v/v). However, the lactic acid yield was always higher than 0.9 g/g and was not influenced by the hydrolysate concentration in the fermentation medium. The secretion of cellulolytic enzymes and the ability to ferment lignocellulose-derived sugars to lactic acid in the presences of inhibitors make this bacterium a suitable starting point for the development of a consolidated bioprocess for the production of lactic acid from lignocellulosic biomass.

## Additional file

**Additional file 1: Table S1.** Media composition. CMC= carboxymethyl-cellulose; SM= Screening Medium; FP= Filter Paper; IM= Induction Medium; BM= Bioscreen Medium; FM= Fermentation Medium. **Figure S1.** Assessment of the optimal pH of growth. **Table S2.** HPLC analysis of filtered microscale cultures with different sugars. **Table S3.** HPLC analysis of filtered microscale cultures in the presence of hydrolysate from acid and steam-exploded wheat straw. sssss

## Abbreviations

CBP: consolidated bioprocessing
CMC: carboxymethyl-cellulose
ddH_2_O: double-distilled water
FP: filter paper
HMF: 5-hydroxymethylfurfural
IM: induction medium
LA: lactic acid
OD: optical density
PLA: polylactic acid
SHF: separate hydrolysis and fermentation
SM: screening medium
SSF: simultaneous saccharification and fermentation
