# Supplementary material for: Bacillus coagulans MA-13: a promising thermophilic and cellulolytic strain for the production of lactic acid from lignocellulosic hydrolysate
Source: Biotechnol Biofuels. 2017 Sep 7;10:210. doi: 10.1186/s13068-017-0896-8 (PMC5590179; doi:10.1186/s13068-017-0896-8)
Supplement: Supplementary file 1 — Additional file 1: Table S1. Media composition. CMC= carboxymethyl-cellulose; SM= Screening Medium; FP= Filter Paper; IM= Induction Medium; BM= Bioscreen Medium; FM= Fermentation Medium. Figure S1. Assessment of the optimal pH of growth. Table S2. HPLC analysis of filtered microscale cultures with different sugars. Table S3. HPLC analysis of filtered microscale cultures in the presence of hydrolysate from acid and steam-exploded wheat straw. [file 13068_2017_896_MOESM1_ESM.pptx]

## Slide 1
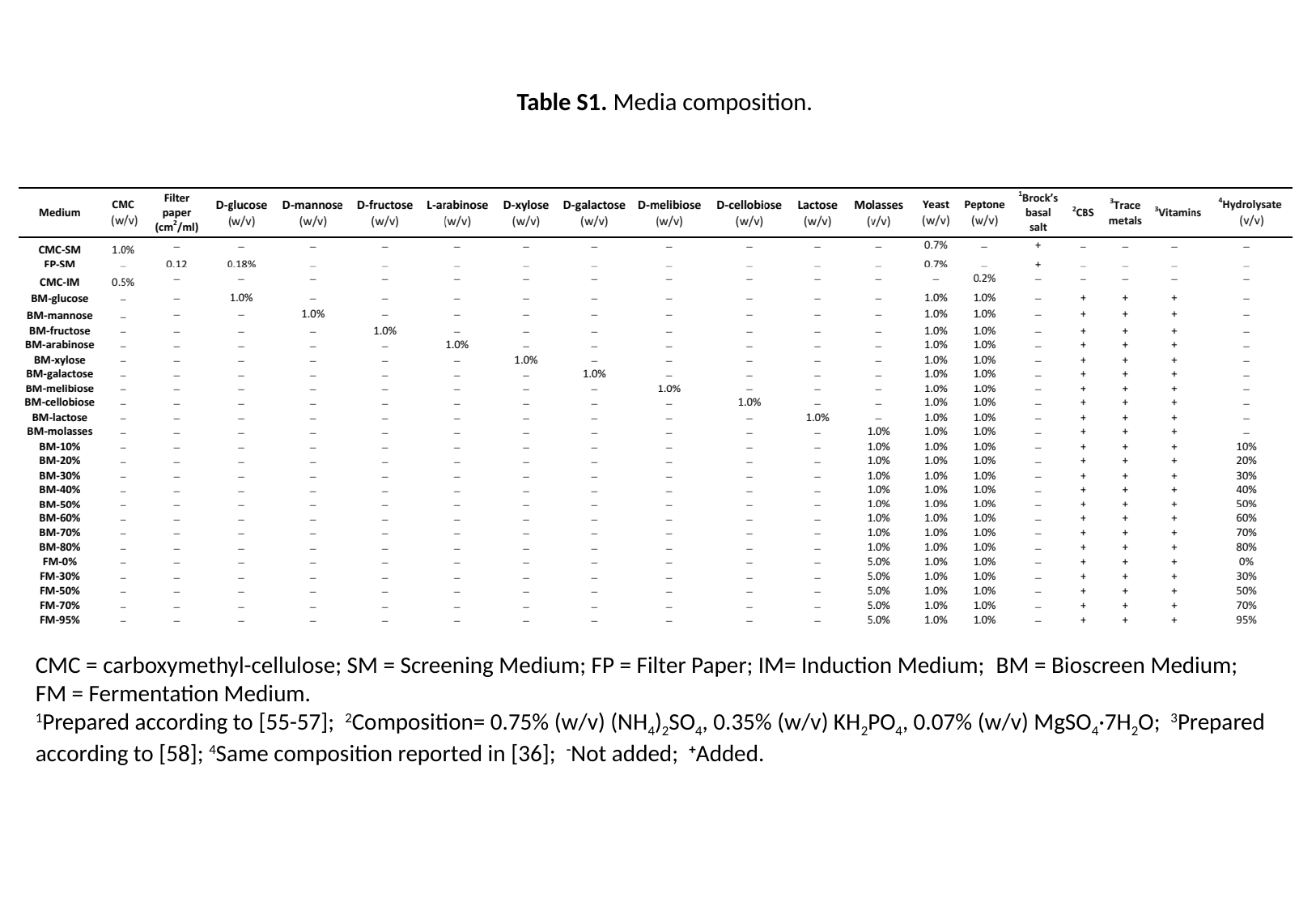

Table S1. Media composition.
CMC = carboxymethyl-cellulose; SM = Screening Medium; FP = Filter Paper; IM= Induction Medium; BM = Bioscreen Medium;
FM = Fermentation Medium.
1Prepared according to [55-57]; 2Composition= 0.75% (w/v) (NH4)2SO4, 0.35% (w/v) KH2PO4, 0.07% (w/v) MgSO4·7H2O; 3Prepared according to [58]; 4Same composition reported in [36]; −Not added; +Added.

## Slide 2
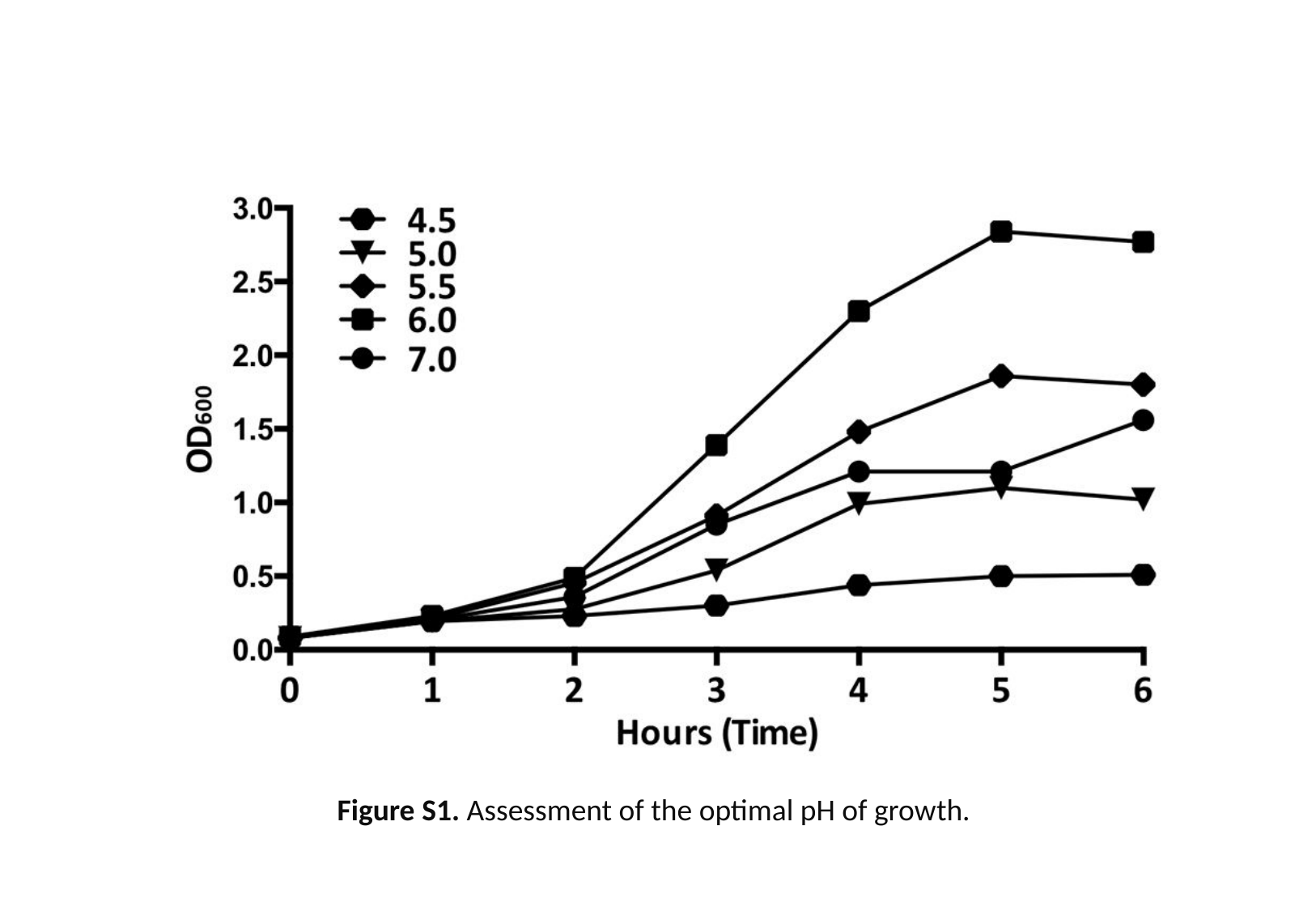

Figure S1. Assessment of the optimal pH of growth.

## Slide 3
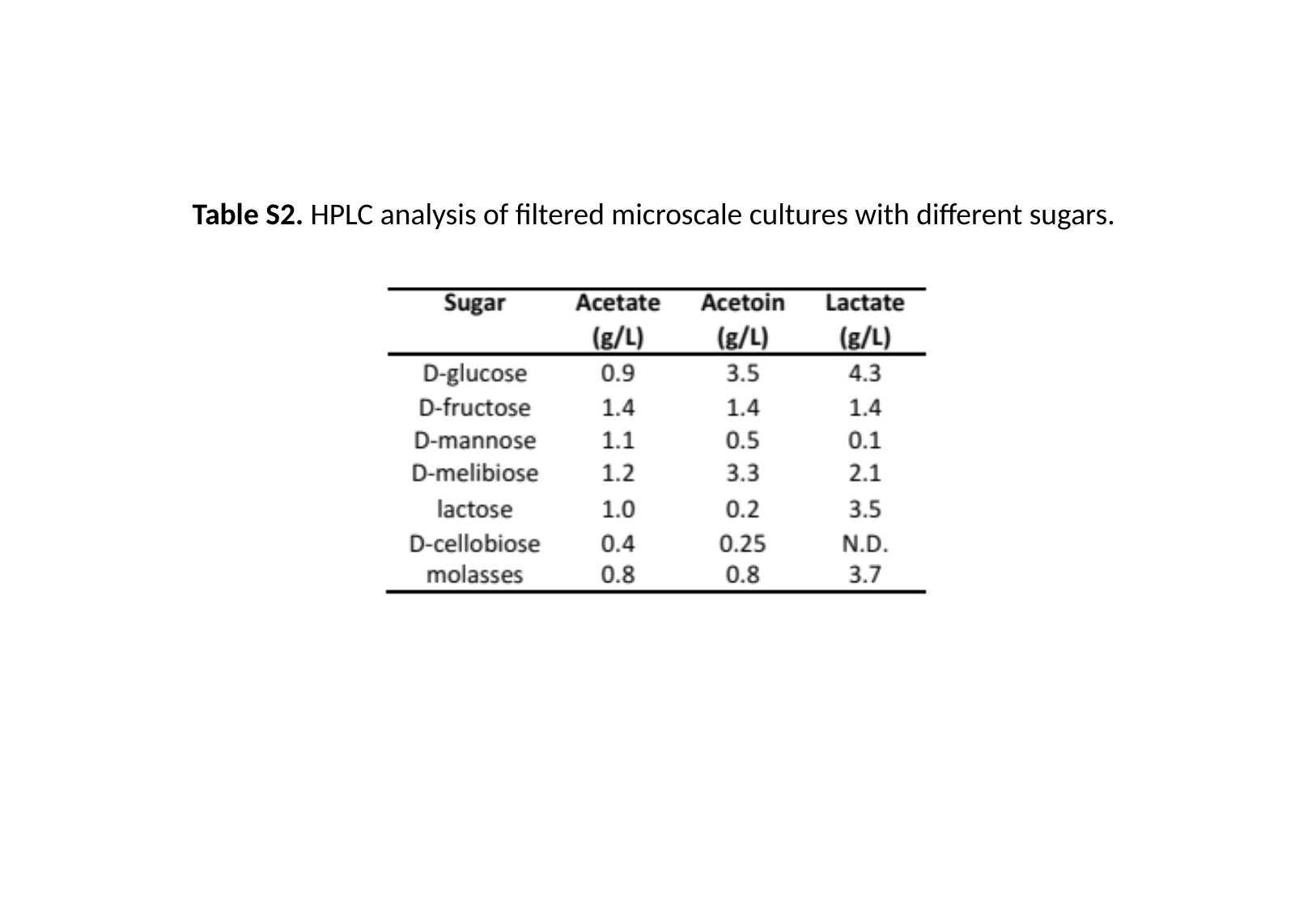

Table S2. HPLC analysis of filtered microscale cultures with different sugars.

## Slide 4
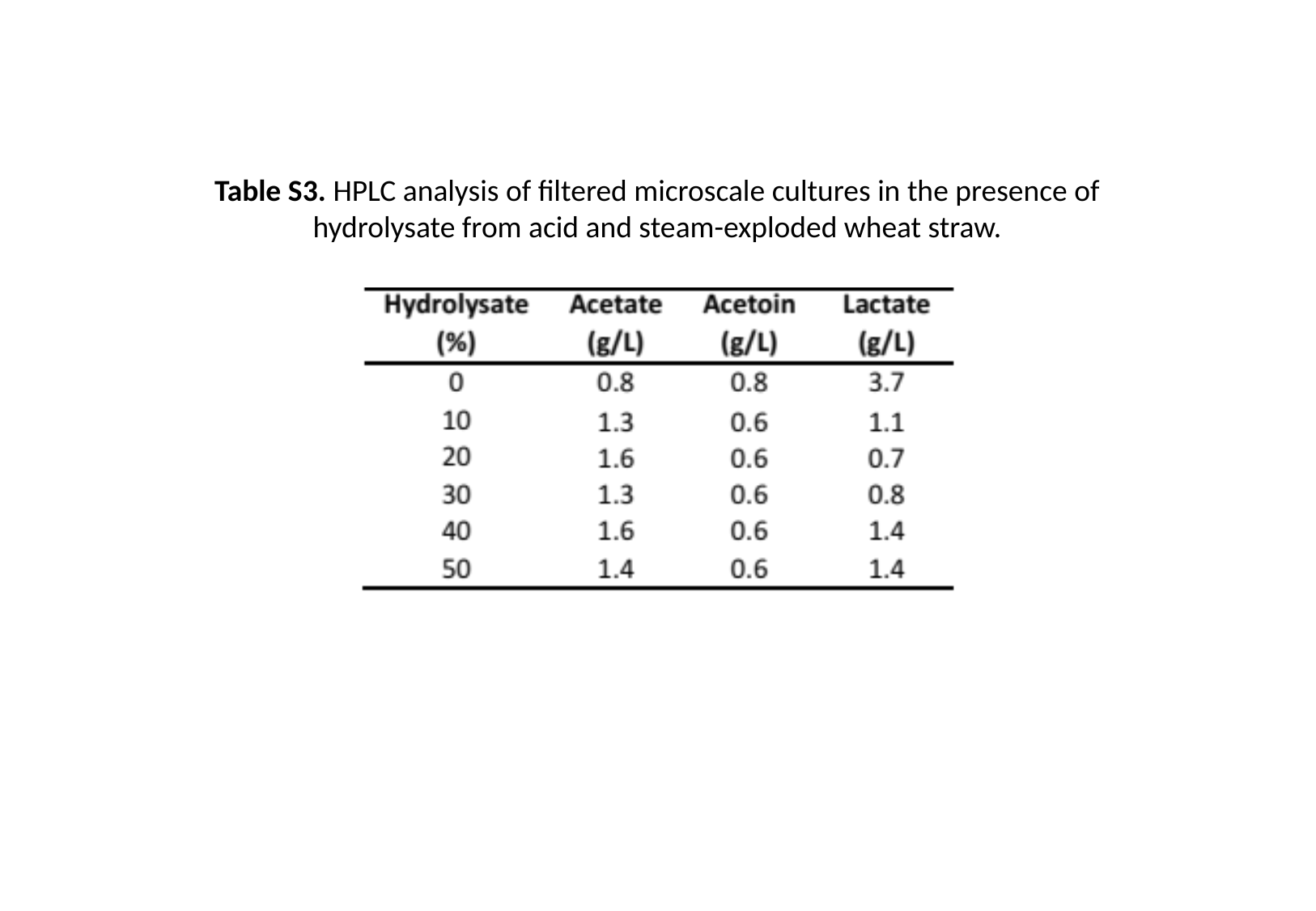

Table S3. HPLC analysis of filtered microscale cultures in the presence of hydrolysate from acid and steam-exploded wheat straw.
